# Editorial

**Published:** 1868-11

**Authors:** 


### Editorial.

TRANSACTIONS OF THE AMERICAN MEDICAL ASSOCIATION.—We have just received the nineteenth volume of the Transactions of our national organization. Like most of the preceding volumes, it is published in excellent style. It contains 497 pages, illustrated with several maps and plates. The contents are made up of the record of proceedings of the Annual Meeting in May, 1868; short reports from the officers of the several sections; the annual address by the President, S. D. Gross; and valuable reports and papers on the following subjects:—Medical Education; Medical Literature; Insanity; Provisions for the Chronic Insane; Topography, Climatology, and Epidemic Diseases of W. Virginia; Climatology and Epidemic Diseases of the District of Columbia; Topography, Meteorology, and Epidemic Diseases of Texas; on the Diseases of Pennsylvania; on the Conveyance of Cholera from Hindostan through Asia, Europe, and America; Plans for the Collection and Statistical Arrangement of Facts, etc.; on Ophthalmology; Treatment of Club-Foot without Tenotomy; New Method of reconstructing the

Lower Lip; New Mode of treating Congenital Talipes; Treatment of Syphilis by Hypodermic Injections; Safe and effectual Operation for the radical Cure of Varicocele; on American Medical Necrology. All these are important topics, and though none of the papers are of tedious length, they possess decided merit.

If any members of the Association, or others, desire a copy of this work and have not secured it by the payment of the annual assessment for 1868, they can still obtain it by forwarding five dollars to Casper Wister, M.D., Treasurer, Philadelphia.

---

Monument to Anæsthesia.—Mr. Thomas Lee, of Boston, sometime since: made a liberal donation for a monument to commemorate the discovery and application of Ether as an anæsthetic in surgical practice.

The work has been completed and placed in the Public Garden, adjoining the Common, in Boston.

It was recently surrendered to the guardianship of the City Authorities by a dedicatory address and presentation by Dr. Henry G. Bigelow.

The following are the proceedings, with a description of the monument, as copied from the *Boston Daily Advertiser*, by a correspondent of the *Cincinnati Lancet and Observer:*—

Mr. Mayor:—It was the wish of the late venerable gentleman, who caused this monument to be erected, to rear an enduring memorial of the discovery in Boston, from which dates the era of painless surgery; and also that on some fitting occasion it should be offered for the acceptance of his fellow-citizens. In no act of a long life, characterized by many deeds of liberality, by the exercise of a refined and cultivated taste of nature and for art, and by a discriminating judgment of men and of passing events, did he show greater discernment, than when he organized this work; and although he did not live to see it executed, he had so far supervised its plans, and so intrusted them to skilful hands, that no difficulty was met in completing its beautiful design in detailed conformity to his wishes.

This monument is intended, in the words of the tablet, which were written since his death, "To commemorate the discovery that the inhaling of ether causes insensibility of pain; first proved to the world at the Massachusetts General Hospital, in Boston, October, A.D. 1846," by its appliance during a protracted dissection, which, when followed by one of the severest operations known to surgery, was a final and conclusive test in a close and connected series of successful experiments, which proved that pain could be annulled: first, with certainty, no matter who the individual; secondly, with completeness, no matter how great might be its degree; and thirdly, with safety. These three points were all absolutely involved in the discovery, and these alone. Before the consecutive experiments which culminated in those here recorded, neither of these points had been established by conclusive proof. The world was ignorant of the great truths they asserted, the discovery had not been made.

The philanthropist had indeed yearned to relieve suffering humanity; the poet had prophetically announced a world free from physical pain; the philosopher had made fruitless efforts to unveil the hidden secret. Instances of accidental insensibility had been observed. Here and there an ingenious man had devised and tried some single experiment with greater or less success, and then abandoned the pursuit; or tantalized by a possibility at one moment in his grasp, and in the next eluding it, stimulated by a flattering promise of achieving something at once practical and useful, had followed up his experiments hopefully, until some great public failure disheartened him, made his proselytes incredulous, and left the world still to suffer pain.

Men had been made insensible to pain through mental excitement, or by the agency of mesmerism or hypnotism, by the dead drunkenness of alcohol, the narcotism of opium, the inhalation of nitrous oxide and other gases, and even by the vapor of ether. For years all this had been known to be possible, but it attracted little attention. These previous experiments, instituted by different persons, were inconclusive, because they led to no constant result; the anæsthesia could not be relied on, or it was not demonstrated that it could be relied on, either sure to occur, or as proof against the severe forms of pain. The question of danger from this extraordinary trance was also unsettled. No consulting board of surgeons would have dared to sanction the production of prolonged unconsciousness during an operation, before the series of consecutive experiments were made here in Tremont St., and at the hospital. There had been a lack of perseverance or of good fortune in the experimenters, or an imperfection in their materials or method, and the future discovery, which was soon to burst upon the world, halted for an interval of years at this imperfect stage. The whole progress of all invention and discovery has been a monotonous catalogue of such imperfect efforts and such failures. But when these consecutive experiments had been made in Boston, the discovery had been made; and in grateful and unhesitating recognition of it, the entire civilized world simultaneously rose up to hail it with acclamatory welcome.

Thus was made the discovery, and thus was begun the career of anæsthetic inhalation. Modifications, imitations, and substitutes have sprung up in all civilized countries. New processes and new materials will yet be furnished by science, or demanded by convenience or economy; but after more than twenty years of its successful trial, nothing has been found to surpass, in its efficiency or unqualified safety, the original ether then used.

To commemorate the triple and demonstrated discovery, not of a probable, an uncertain or untrustworthy, but of an inevitable, complete, and safe anæsthesia, this monument has been erected in a city which was the humble instrument of Divine Providence in diffusing to the nations this incalculable blessing.

I well remember when the eloquent and gifted man, whose brazen effigy on yonder pedestal so powerfully recalls his living presence, in an address delivered at the Medical College on the 4th of November, 1846, said, with an unconscious foreshadowing of what was soon to happen:—"I cannot suppress the remark that the great principle of analogy seems to authorize the hope that * * * further discoveries may be expected scarcely less brilliant than that of vaccination." How even far this prophetic inspiration fell short of the reality! How little did he dream that the lapse of a few brief days would herald to the earth the greatest boon ever accorded to the physical welfare of mankind; days of discovery that forever silenced the dreadful shriek of agony which many of us can yet recall in the surgical amphitheatre of the institution whose name is now immortalized, that stilled the moan of the soldier stricken down upon the battlefield, assuaged the pangs of disease, softened the approach of death, and lent a sweet obliviousness in what was once its hour of anguish to all animal existence, from the poor suffering brute up to humanity, to man born of woman, and to woman of whom man was born.

In the name and at the request of my venerable friend, the late Mr. Thomas Lee, of his executors, and of the gentlemen to whom he intrusted the arrangement of this ceremony, I beg to offer this memorial to you, sir, and through you to the city of Boston.

The remarks of Dr. Bigelow elicited considerable applause. Mayor Shurtleff responded as follows:—

In behalf of the municipal authorities of Boston, I now formally receive from you the gift of Mr. Thomas Lee; and promise that it shall be watched with care and protected from injury. And may this elegant structure long remain unimpaired by time, a memorial of the greatest boon ever vouchsafed to suffering humanity, and a monument of the gratitude of one of Boston's most worthy citizens.

The exercises were closed with an appropriate prayer by Rev. Dr. Lothrop, and after lingering about the monument for a while to admire its beauty and fine proportions, the audience dispersed.

We append a description of the monument, copied from the "History of the Water Works," by Nathaniel J. Bradlee, Esq., President of the Cochituate Water Board.

The form of the monument is suggested by mediæval types, modified by the nature of the white Concord granite used in its construction. It is about thirty feet in height, and arises from a square basin. Its base is cubical, leaving on each vertical face a niche containing a spouting lion's head, with sculptured water-lilies and other aquatic plants. Upon this base or plinth rests a surbase, adorned with mouldings from which arises a die, bearing upon each of its four sides an inscription, surmounted by a bas-relief in marble. These are sunk in the tympana of four pointed and cuspidated arches, supported each by two stunted shafts of red Gloucester granite, the capitals of which are enriched by poppies and oak leaves, this decoration being carried around the monument on the same level in a band or string course.

These arches form a canopy, square in plan, from which the structure diminishes by a series of mouldings to the base of a grouped quadripartite shaft of polished red granite. Its capital, which is decorated with oak leaves, bears on its abacus a group setting forth the story of "the good Samaritan," the type of the relief of suffering.

The inscriptions and bas-reliefs on the four sides are successively as follows:—

I.

To commemorate
the discovery
that the inhalation of ether
causes insensibility to pain.
First proved to the world
at the Mass. General Hospital
in Boston,
October, A.D. MDCCCXLVI.

The bas-relief accompanying this represents a surgical operation in a civic hospital, the patient being under the influence of ether.

II.

Neither shall there be any more pain.

[Revelation.

With an allegorical bas-relief of the Angel of Mercy descending to relieve suffering humanity.

III.

In gratitude
for the relief
of human suffering
by the inhaling of ether,
a citizen of Boston
has erected
this monument,
A.D. MDCCCLXVII.

With a bas-relief of a field hospital, with a wounded soldier in the hands of the surgeons.

IV.

This also cometh forth
from the Lord of Hosts,
which is wonderful
in council
and excellent
in working.

[Isaiah.

The bas-relief accompanying this inscription is an allegory of the triumph of science. B.

---

Mortality Report for the Month of September:—

The monthly report for September showed 133 deaths from cholera infantum, 78 from convulsions, 44 from diarrhœa, 22 from dysentery, 29 from typhoid fever, 15 from hydrocephalus, 39 from phthisis pulmonalis, 27 from tabes mesenterica, 30 from teething, and 10 from teething and diarrhœa. The total mortality was 736; 382 males, 354 females; 108 married, 628 single; 9 colored, 727 white. In September of last year the deaths were 507, an increase over this year of 229. In August last the total was 944, a decrease of 208.

The highest mortality was of children under one year, 277; one year to three, 218; between 20 and 30 years, 49.

The nativities were: Africa, 1; Austria, 1; Bermuda, 1; Bohemia, 7; Canada, 6; Chicago, native, 158; foreign, 28; United States, other parts, 107; Denmark, 1; England, 9;

France, 1; Germany, 70; Holland, 4; Ireland, 52; Italy, 3; Norway, 10; New Brunswick, 1; Scotland, 1; Sweden, 17; Wales, 1; unknown, 1.

The highest percentage of deaths in the wards was in the Seventh, being 112, one in 195 of population; the lowest in the First ward, being 9, or one in 1332. There was 1 death in Bridewell; County Hospital, 12; Home for the Friendless, 44; Half-Orphan Asylum, 1; Hospital of the Alexian Brothers, 1; Marine Hospital, 2; Mercy Hospital, 3; Protestant Orphan Asylum, 1; Immigrants, 19.

The deaths in the several wards during September were: First, 9; Second, 25; Third, 27; Fourth, 36; Fifth, 42; Sixth, 46; Seventh, 112; Eighth, 48; Ninth, 34; Tenth, 38; Eleventh, 33; Twelfth, 74; Thirteenth, 44; Fourteenth, 43; Fifteenth, 64; Sixteenth, 26.

THE DOG NUISANCE.

The inspectors' reports showed that during the month, 963 dead dogs had been removed in the South Division; 1615 in the West Division; and 465 in the North Division; total, 3043.

---

THE OPHTHALMOSCOPE IN DISEASES OF THE NERVOUS SYSTEM.—M. Bouchut has just sent to the Academy of Sciences, of Paris, the results of his more recent researches on the utility of the ophthalmoscope in diagnosticating diseases of the cerebro-spinal system. Through the novelty and interest of the subject we are induced to sum up briefly the more striking features of this memoir. Most of the diseases of the membrane of the brain and spinal cord being accompanied by optic neuritis, neuro-retinitis, inflammation of the choroidal membrane and papillary atrophy, it can be understood how the ophthalmoscope enables us to detect in the interior of the eye disorders of circulation, of secretion, and of nutrition, which indicates an organic disease of the cerebro-spinal system. It is through the anatomical and physiological connections of the eye with the spinal cord and brain, that we may explain the law of coincidence of optic neuritis with organic injuries of the nervous system. Each time that some violent impediment to cerebral circulation is brought on by the existence of some injury of the cerebrum and of the spinal cord, papillary and retinal hyperæmia is the consequence.

When it is the brain which is the seat of acute or chronic phlegmasia, the inflammation may extend to the eye by following the course of the optic nerve. On the other hand, diseases of the anterior columns of the cord may, through the anastomosis of the parts with the great sympathetic nerve in the situation of the two first dorsal pairs, produce in the eye various phenomena of papillary hyperæmia, which brings on, at a subsequent period, wasting of the optic nerve.

These facts show through what mechanism diseases of the nervous system stamp themselves on the eye so as to be detected by the ophthalmoscope. Other results are mentioned by the author, which may be of use whilst determining the diagnosis. Thus the optic neuritis and neuro-retinitis, produced by the acute or chronic diseases of the nervous system, are generally observed in both eyes; in cases of injury of the brain, or its membrane, optic neuritis is habitually more marked in the eye corresponding to the hemisphere which is more seriously altered; changes of the optic nerve and retina, complicated by disorders of sensibility, intellect, and movement, invariably indicate an organic disease of the encephalon. It may be added, that the alterations of the optic nerve and the retina should not be isolated from the other symptoms of the morbid condition. When considered thus, detection of their presence gives to the diagnosis an undeniable certitude.

The author concludes by naming the diseases of the nervous system in which optic neuritis and neuro-retinitis are observed, and he draws up the following list:—Phlebitis of the sinuses, acute or chronic meningitis, chronic encephalitis, cerebral hemorrhage, tumors of the brain, contusion and compression of the brain, chronic hydrocephalus, abscess of the brain, acute myelitis, locomotor ataxy, essential or idiopathic contraction, and certain cases of epilepsy, of paralysis, or of neurosis, associated with an organic lesion of the nervous substance.—*Lancet.*

---

Nasal Therapeutics.—At a recent meeting of the Liverpool Medical Institution, reported in the *British Medical Journal*, Dr. Banks, one of the members, made the following extremely practical remarks on the Application of Remedies to the Nostrils and Larynx:—

"Weber, of Leipzic, discovered fifty years ago that when a column of water was passed along one nostril, when it touches the soft palate, it causes it to rise so as to shut off the nasal from the pharyngeal cavity, so that the fluid is compelled to return through the other nostril. Weber, of Halle, first put the principle into practice; and in 1864, Dr. Thudichum invented an instrument, and published some papers on the subject. The points to be attended to in using the instrument were the following: 1, the nozzle should fit the nostril accurately; 2, in children and nervous people, the full stream should not be turned on at once; it should be allowed to pass in gently at first, and then gradually increase in volume and force; 3, the current should be reversed occasionally. Cold water is irritating; and, therefore, tepid water, or a solution of an ounce of salt in a pint of water, may be used, followed by a deodorizer, as Condy's fluid, liquor carbonis detergens, or especially carbolic acid, and afterwards a stimulant astringent, as alum (one drachm to the pint), sulphate of zinc or copper (from ten to thirty grains to the pint), etc. The solution should not be too strong at first. The instrument was also useful in some surgical accidents, such as a foreign body in the nostril and severe epistaxis, when some dilute hæmostatic should be employed. Dr. Skinner had been practically making use of this principle before Dr. Thudichum's paper appeared, the instrument employed being a Higginson's syringe. The author had found Mr. Bryant's mode of treating nasal polypi, by blowing tannin into the nostrils through a quill, very satisfactory in some cases, especially soft and gelatinous polypi. Another troublesome affection—a chronically swollen and thickened condition of the nasal and palatine mucous membrane—was benefited by the administration of iodide or bromide of potassium; but local astringents were also useful, and were best applied by means of the spray producer. The best applications were, glycerine of tannic acid (one scruple to one ounce of water), or a solution of iodine, with a small quantity of carbolic acid. Speaking of affections of the throat, the author observed, that of the various instruments devised to bring remedies into contact with the air-passages, the spray producer was the best. Its use was very great in chronic laryngeal affections, and also in many acute affections, as putrid sore throat and scarlatinal cynanche, diphtheria. The spray producer could not be employed with very potent remedies, such as strong solution of nitrate of silver. A piece of whalebone, bent at an obtuse angle near the end, and having a brush (better than a sponge) attached to it, was the best instrument for applying these. Care and dexterity are requisite in using it."—*Med. and Surg. Reporter.*

---

The Use of Pepsine in Infantile Diarrhœa.—James S. Hawley, M.D., of Greenpoint, L. I., advocates the use of *American*

*pepsine* in the diarrhœa of infants, for the purpose of converting the ingesta into nutriment. The food going through the intestinal canal in an undigested form, becomes an irritant. And this is not all: the food does not always remain a simple, foreign substance, but undergoes putrefaction, decomposition adding new and more active sources of disease.

The indications in this disease are as follows:—*First*, to remove all sources of irritation from the quality of the ingesta; *Secondly*, allaying irritation by sedatives; *Thirdly*, artificial digestion by the administration of pepsine.

M. Corvisart and M. Barthez, of Paris, have made use of pepsine, both in adult and infantile cases, with success.

Dr. H. has been in the habit of administering pepsine in the diarrhœa of fed and teething infants for several years, with favorable results.

Cases are detailed by him, where the following simple prescription terminated the disease:

℞. Am. Pepsine, Subnit. Bismuth, āā gr. v. every three or four hours.

℞. Am. Pepsine, Subnit. Bismuth, āā ʒj.; Pulv. Opii, gr. j.; divided into twelve powders, and one given every two to four hours, according to circumstances.

In conclusion, he commends pepsine in the treatment of infantile diarrhœa, especially during the period of dentition. It has no noxious or perturbing qualities, and to some extent has borne the test of experience.

---

Division and Excision of Nerves.—Some months since, a case occurred in Paris, which attracted much attention, and caused considerable speculation. A woman received a wound just above the wrist, which completely divided the median nerve, notwithstanding which the peripheral end of the nerve was painful when touched, and the fingers retained the property of feeling. Some observers sought to explain the retention of sensibility by supposing an anomalous distribution of nerves, but Professor Richet believed the painful impression produced by touching the peripheral extremity of the nerve to be due to the presence of *nervi nervorum*, lately discovered by M. Sappey; and that the retention of sensibility by the fingers was in consequence of the corpuscles of tact being supplied by filaments from the radial and ulnar nerves, as well as the median, as demonstrated by Cruveilhier and Robin. In consequence of the interest manifested in the above case, Dr. J. F. Miner has been induced to record in the June number of the *Buffalo Med-*

*ical and Surgical Journal*, three others of a similar nature, which have occurred in his practice. A man received a gun-shot wound of the arm, the ball passing directly through the median nerve. False anchylosis eventually ensued, and the pain from the receipt of the injury was intense and indescribable. Twenty-two weeks from the time of injury three inches of the nerve, which was found closely involved in the cicatrix, were removed, the pain ceasing immediately. *Sensation* was at no time lost, but was at first somewhat diminished along the anterior portion of the forearm and hand.—The second case was one of cystic tumor of the arm, which had existed five years, latterly becoming exceedingly painful. It so involved the musculo-spiral nerve that the removal of the tumor necessitated the removal of a portion of the nerve. This was followed by loss of motion, and in great degree of sensation, of all the parts supplied by the nerve. In the third case, a wound dividing all the flexor tendons of the middle, ring, and little fingers, the ulnar artery and nerve, *sensation* was almost entirely lost immediately after the injury, but was afterwards perfectly regained, the wound healing rapidly and the nerve, of course, uniting.

---

Present State of Health in London.—It is stated that (*Lancet*, July 11, 1868) there is a rapid and serious increase of diarrhœa in London, and other large towns. In the week ending June 6th, the deaths from this cause in London were only 27. In the four following weeks they were 31, 66, 171, 286. In the earlier weeks of the present unusual heat, according to Dr. Glover, the most noticeable effect upon health was a degree of asthenia, with more or less discomfort in the stomach and bowels. Diarrhœa had not shown itself to any serious extent. The mortality of London and the large towns was very low. It, has, however, gone rapidly up. The mortality, instead of being 19 per 1000, was last week at the rate of 25 in London, 26 in Liverpool. Of the 577 deaths from zymotic diseases in London, 286 were from diarrhœa, 19 from "cholera," or choleraic diarrhœa. Of the 286 persons, 230 were children under one year of age. It is probable that we have not seen the full development of this complaint, unless, indeed, we have an unlikely change in the weather. The meteorological character of the season is such as to throw light on the causation of diarrhœa. A very high temperature and extreme drought are conditions that generate this complaint on an epidemic scale. The fruit theory of its causation is not tenable: witness the age of its principal victims.

Health of London.—During the last week in July, the mean temperature of the air was 69.2°, or 7.6° above the average of the corresponding week in 50 years, according to Mr. Glaisher's observations. If anything, there was a little less heat than in the week preceding, but the difference was not sufficient to avert the unfavorable influence which great heat combined with continued drought always exercises on the populations of great cities. The registered deaths amounted to 1885 in the week, or more by 243 than were recorded the week before, and 310 more than the weekly average of the season. The deaths from diarrhœa have risen from 340 to 442, and by the more serious choleraic form of disease from 37 to 58. It is to be noticed that the mortality from diarrhœa, unlike that from true cholera, is pretty equally diffused through the various water-fields of London. Zymotic disease caused 800 deaths, or 43 per cent. of the total mortality, the corrected average being only 631 deaths from this class of causes. Of the 58 deaths returned under the head of "cholera," 38 were of children under 1 year of age, 8 aged 1 year, and 4 between 2 and 5 years, leaving 7 cases of adult death resulting from attacks of cholera not exceeding thirty hours' duration.—*Lancet*, August 1, 1868.

---

Personal Appearance of Virchow.—A correspondent of the *Cincinnati Lancet and Observer*, writing from Berlin, gives the following description of the great pathologist: Instead of the commanding or inspiring physique we had anticipated, we beheld a small man, with a short, quick, determined step, giving a nervous twitch to his whole body in progression; a face stamped strongly with the national characteristics, but chastened by hard study; a forehead expanded in every direction, with the lines that deep thought alone produces; a peculiar glance, such as you have often noticed from eyes accustomed to microscopic research, lending rather a furtive expression which is perhaps increased by the spectacles which he constantly wears; delivery slow, distinct, the purest German, no jingle of rhetoric, scarcely a gesture; merely a dictum which the lecturer deposes as law, without being at all dogmatical, and which is law.—The writer adds that he lectures daily from five to seven hours, on medicine "and politics," and that he is an earnest and powerful advocate of the liberal party, and the chief of the opposition to the divine right of kings.

---

Tobacco a Cause of Bald Heads and Gray Hair.—Dr.

D. B. Hoffman states (*Pacific Medical and Surgical Journal*, June, 1868) that a large proportion of the young men in California are bald-headed and have gray hair, which is not the case with females. This he is inclined to attribute to the excessive use of tobacco by the men. In support of this view he relates the case of one of his patients, under forty years of age, who had been in the habit of using tobacco to excess, and had been for 5 or 6 years both bald-headed and gray-haired. With great resolution he abandoned the use of tobacco: the result was that he entirely recovered his health, which had been bad; his whole head had become covered with a luxuriant growth of fine black hair, and he lost the sallow, beeswax hue of skin so common in those who use tobacco to excess.

---

DEATH FROM CHLOROFORM.—Mrs. Elizabeth Hammon, aged 35, mother of three children, apparently a healthy woman, went to a dentist (Dr. McDowell) April 16, 1868, for the purpose of having some teeth extracted. Thinking she could not endure the pain, she requested the doctor to administer chloroform; and, as he had given it to her once before (about six months since) without any bad effect, he consented to administer it again, and, pouring about two drachms of chloroform upon a sponge, held it a short distance from her face. After she had made three or four inspirations her respiration ceased; he felt for the pulse and found she had none. Artificial respiration was commenced at once, and stimulating applications applied over the heart and to the extremities, but to no effect. She made but two or three efforts at inspiration after he first noticed something was wrong. An autopsy could not be obtained.

---

THE EARLY HISTORY OF SYPHILIS IN CHINA.—Dr. Geo. Thin, of Shanghai, China, contributes to the *Edinburgh Medical Journal* some interesting historical notes on this subject. He was assured by many Chinese scholars that syphilis has been known to exist in China for many centuries, and he therefore undertook, with the assistance of a learned native antiquary, to hunt up the records. He finds that in the seventh century the venereal chancre was described under a specific name, which places its nature beyond a doubt, and that from this time onward there are various allusions to it, although in modern times the more ancient notices have in a great measure been overlooked, partly from change of nomenclature, and partly from the fact that the works in which the notices occur are not likely to come before the general practitioner. Even anterior to the

Christian era, there are many traditions and vague references, which are generally accepted as indicating syphilitic diseases. The earliest of these is to be found in a collection of odes made by Confucius five hundred years B.C.—*New York Med. Jour.*

In consideration of the numerous victims of homœopathic treatment, a decree of the Emperor of Russia prohibits the practice of homœopathy in the entire territories of Russian America.—*Union Medicale.*

MEDICAL FACULTY OF PARIS.—M. Denonvilliers, Professor of Operative Medicine, has been transferred to the Chair of Clinical Surgery in this faculty, in place of M. Jarjavay, lately deceased.

